# Path analysis of cardiovascular disease preventive health behavior in postmenopausal Korean women based on the I-Change model

**DOI:** 10.1038/s41598-026-55974-z

**Published:** 2026-06-13

**Authors:** Se Jin Hong, Sooyeon Park

**Affiliations:** 1 College of Nursing, Research Institute of AI and Nursing Science, Gachon University, Incheon, Republic of Korea; 2 College of Nursing, Konyang University, Daejeon, Republic of Korea

**Keywords:** Cardiovascular disease, Health behavior, I-Change model, Postmenopausal women, Diseases, Health care, Medical research, Risk factors

## Abstract

Postmenopausal women are at increased risk of cardiovascular disease (CVD) owing to hormonal changes; however, many remain unaware of this increased risk and do not engage in CVD-preventive health behavior. This study aimed to examine factors associated with CVD-preventive health behavior among postmenopausal women living in residential area across South Korea. Data were collected through an online survey of 227 community-dwelling postmenopausal women. Associations among CVD knowledge, risk perception, attitudes, social support, intention, and CVD-preventive health behavior were examined using the Integrated Change (I-Change) model and analyzed through path analysis. The path analysis showed that CVD knowledge had a direct effect on attitudes (β = .21, *p* < .001), self-efficacy (β = .15, *p* = .021), and social support (β = .15, *p* = .023). Self-efficacy (β = .37, *p* < .001) and intention (β = .38, *p* < .001) had direct effects on CVD-preventive health behavior. The final model explained 48.7% of the variance in CVD-preventive health behavior. These findings suggest that self-efficacy and intention play important mediating roles in the association between CVD knowledge and CVD-preventive health behavior among postmenopausal women. Therefore, interventions aimed at reducing CVD risk should consider strategies to enhance self-efficacy and strengthen behavioral intention.

## Introduction

Cardiovascular disease (CVD) is a leading cause of morbidity and mortality worldwide, affecting quality of life and imposing a substantial socioeconomic burden^1^. In South Korea, the CVD mortality rate has steadily increased over the past decade, reaching 134.7 per 100,000 population in 2024. Notably, the mortality rate is higher in women (138.6 per 100,000 per year) than in men (130.7 per 100,000 per year), particularly among postmenopausal women aged over 50 years^2^. This increased risk is largely attributed to menopause-related physiological changes^3^.

In addition to these physiological factors, lifestyle-related risk factors may further contribute to the elevated risk of CVD. Korean women tend to have relatively low levels of physical activity and high sodium intake, both of which are known to increase cardiovascular risk. National statistics indicate that only 13.8% of women aged 45–64 years engage in regular physical activity, and their sodium intake remains substantially higher than the recommended level^4,5^. These lifestyle-related factors may have greater implications when combined with menopause-related physiological changes, further exacerbating cardiovascular vulnerability.

Menopause, defined as the permanent cessation of menstruation due to decreased ovarian function, induces significant hormonal and metabolic changes. The decline in estrogen levels contributes to dyslipidemia, insulin resistance, and hypertension, all of which increase the risk of CVD^6^. Therefore, postmenopausal women represent a particularly vulnerable population in whom physiological and behavioral risk factors interact to increase cardiovascular risk. Therefore, the American Heart Association has classified postmenopausal women as a high-risk group and has provided specific guidelines for CVD prevention^7^. However, despite these recommendations, many women remain unaware of their increased risk and do not engage in adequate health-promoting behavior^3^.

Previous research has shown that risk perception and CVD knowledge among women have declined globally, including in South Korea^8,9^. This lack of awareness may hinder the adoption of health-promoting behavior, particularly in populations already at elevated physiological risk. Given the increasing life expectancy of Korean women, improving cardiovascular health among postmenopausal women is essential for promoting healthy aging and reducing the burden of chronic disease^3^.

Encouraging health-promoting behavior is a key strategy for reducing CVD risk^10,11^. In this context, understanding the behavioral determinants of such actions is crucial. Prior studies have identified factors such as CVD knowledge, risk perception, self-efficacy, and social support as important influences on CVD-preventive health behavior^12–16^. However, findings have been inconsistent, and most studies have examined these factors in isolation. Limited research has explored how these variables interact within a comprehensive framework, particularly in high-risk groups such as postmenopausal women^17,18^.

To address this gap, theoretical models of health behavior change can provide a useful framework. The Integrated Change (I-Change) model offers a comprehensive approach by incorporating cognitive, motivational, and social-environmental determinants of behavior. Recent studies suggest that key constructs of the I-Change model, including knowledge, self-efficacy, social support, and intention, play an important role in promoting lifestyle changes such as diet, physical activity, and smoking cessation. Although these studies did not explicitly adopt the I-Change model, their findings support its theoretical framework by demonstrating the importance of these factors in influencing cardiovascular health behavior^19–21^. Although widely used in intervention studies, its application to CVD-preventive health behavior in Asian populations remains limited. Thus, a theoretical framework that integrates these multidimensional factors is needed to better understand behavioral processes in postmenopausal women.

Therefore, this study aimed to apply the I-Change model to identify key behavioral determinants of CVD-preventive health behavior among postmenopausal women in Korea and to provide a basis for developing evidence-based interventions.

### Conceptual framework

The I-Change model provides a conceptual framework for understanding health behavior change by integrating cognitive, motivational, and social-environmental factors. It conceptualizes behavior change as a process occurring across three phases: pre-motivational (awareness), motivational (motivation), and post-motivational (action), each influenced by distinct determinants^22^.

Given the increased cardiovascular vulnerability of postmenopausal women due to the combined effects of physiological changes and lifestyle-related factors, a comprehensive framework is needed to explain how multiple determinants jointly influence health behavior. The I-Change model was selected because it allows for the systematic integration of these multidimensional factors and facilitates a more comprehensive understanding of health-promoting behavior.

In the I-Change model, the pre-motivational phase involves the development of awareness through factors such as knowledge and risk perception. The motivational phase focuses on the formation of behavioral intention, shaped by attitudes, social influences, and perceived self-efficacy. The post-motivational phase refers to the execution of health-promoting behavior.

**Fig. 1:**
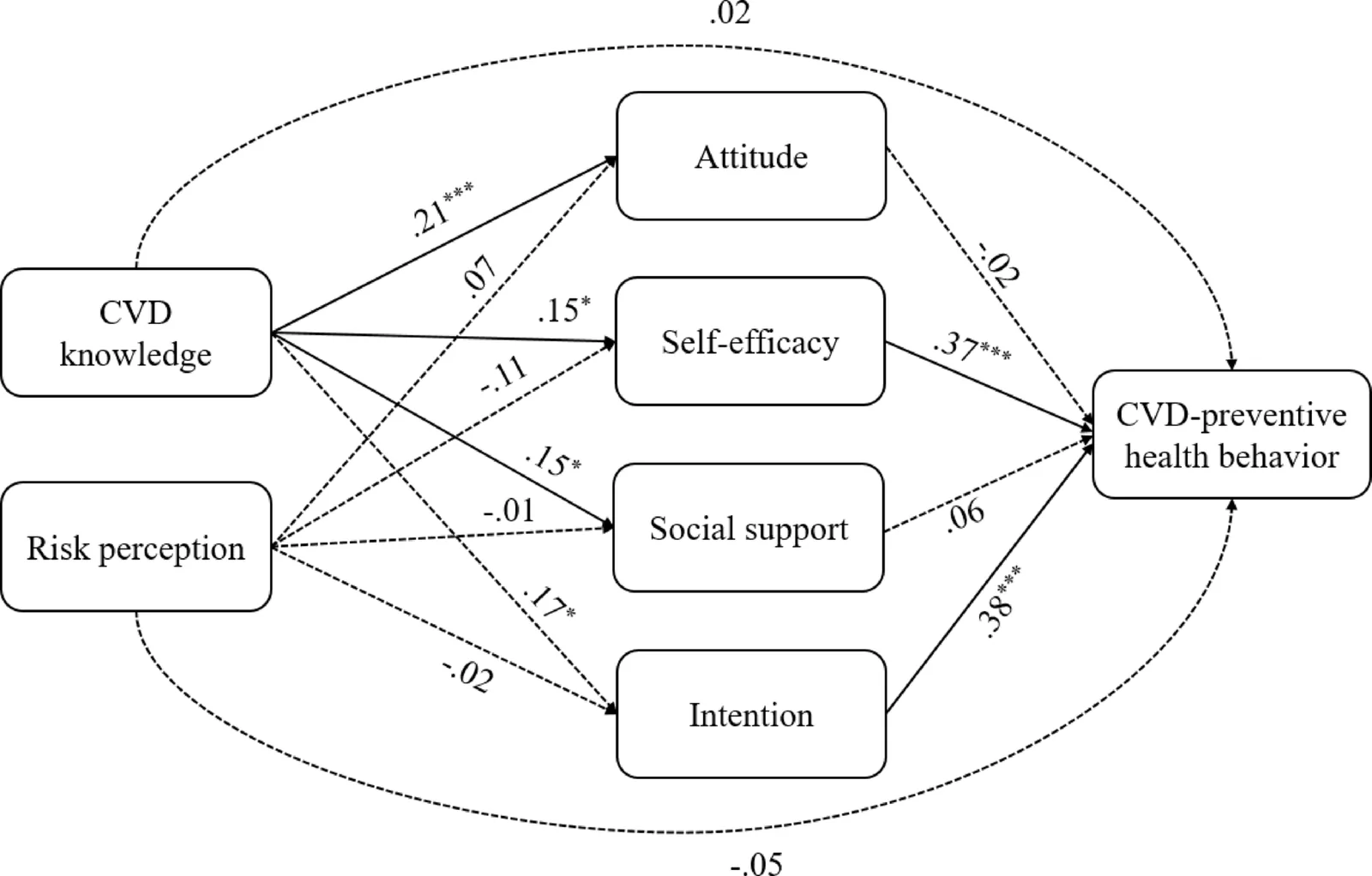
Results of the path analysis showing standardized estimates of the final model. *Note* Solid lines = significant paths; Dashed lines = non-significant paths. Numbers are standardized coefficients (β). *p < .05, ***p < .001.

In this study, the I-Change model guided the development of a hypothetical path structure to examine CVD-preventive health behavior among postmenopausal women. Variables were selected based on the model to represent each phase of behavior change and to reflect key determinants identified in previous research. These determinants include knowledge of CVD risk factors and risk perception (awareness); attitudes, self-efficacy, social support, and intention (motivation); and actual CVD-preventive health behavior (action) (Fig. 1).

We hypothesized that: (1) CVD knowledge and risk perception would be positively associated with attitudes, self-efficacy, social support, and intention; (2) attitudes, self-efficacy, social support, and intention would be associated with CVD-preventive health behavior; and (3) these motivational factors would mediate the relationship between awareness and action.

## Methods

### Design and participants

This study used a cross-sectional survey design. The participants were community-dwelling postmenopausal women residing in nationwide residential areas who were recruited through a survey panel managed by a professional research company. This company maintains a nationwide panel of 1.3 million individuals who have been pre-screened and verified for demographic accuracy. Furthermore, to ensure the representativeness of the panel, its composition is aligned with population estimates from the national statistical office. To enhance the validity of the study, quota sampling based on age and region was employed, and duplicate responses were prevented through IP and device tracking. Screening questions were used to identify and select respondents who met the eligibility criteria from among the panel members. The survey was conducted in June 2024. All participants had provided prior consent for participation in surveys during the panel registration process and received instructions for participating in the survey via email. The specific inclusion criteria were: (1) women aged 50–64 years; (2) those who had achieved natural menopause; (3) those whose last menstrual period had occurred more than 1 year prior; and (4) those who understood the purpose of this study and provided voluntary consent to participate in the survey. The exclusion criteria were (1) having received female hormone therapy within the past year and (2) being diagnosed and treated by a physician for a serious chronic illness (e.g., cancer). The number of participants in this study was based on an appropriate sample size for maximum likelihood methods, which is at least 200, or 10 times the number of observations per variable^23^. The minimum required sample size was 180 based on a minimum of 18 variables, and 230 participants were selected to account for a dropout rate of at least 20%. Of the 230 respondents, 227 were included in the analysis after excluding three respondents who did not complete all questions on the questionnaire.

### Measurements

#### CVD knowledge

To determine the knowledge of CVD symptoms and risk factors, we adapted items from the AHA and regional cardiovascular center training manuals and applied the tools used by Choi and Kim^24^ in their study on a CVD prevention education program for postmenopausal women. The symptom and risk factor knowledge tools comprised 10 and 6 questions, respectively.

Representative items include: “Nicotine in cigarettes increases blood pressure and heart rate” and “Long-term consumption of salty foods can increase blood pressure.” Each question was scored 1 point for correct answers and 0 for unknown or incorrect answers. The higher the score, the greater the knowledge of CVD symptoms and risk factors. In the study by Choi and Kim^24^, the reliability for symptom and risk factor knowledge, using the Kuder-Richardson 20 (KR-20) formula, was 0.74 and 0.82, respectively. In this study, the KR-20 scores for symptom and risk factor knowledge were 0.70 and 0.61, respectively.

#### Risk perception

Risk perception was measured based on a Korean translation of the CVD risk perception scale developed by Becker and Levine^25^. CVD risk perception refers to an individual’s subjective assessment of their likelihood of developing CVD. To ensure an instrument’s content accuracy, a formal translation and back-translation process was conducted by two nursing scholars. Furthermore, the Content Validity Index was assessed by nursing experts and demonstrated a value exceeding 0.8. The risk perception scale consists of four questions and is scored on a 6-point Likert scale ranging from 1 (never, not likely, very low) to 6 (always, most likely, very high). Representative items include: “How often do you worry about having a heart attack?” and “How likely do you think you are to develop cardiovascular disease within the next 5 years?” The overall score was calculated as the mean of the item scores, ranging from 1 to 6, with higher scores indicating a higher perceived CVD risk. Cronbach’s α was 0.86 both in the study by Hwang et al.^26^ and in the present study.

#### Attitudes

Attitudes toward CVD prevention were measured using an 8-item questionnaire focusing on content on healthy lifestyles to prevent CVD developed by the Korean Ministry of Health and Welfare and eight CVD-related societies^27^. This instrument reflects individuals’ cognitive evaluations and beliefs regarding the effectiveness and importance of specific CVD-preventive health behavior. Representative items include statements such as “Quitting smoking can help prevent cardiovascular disease” and “Engaging in regular physical activity can help prevent cardiovascular disease.” Responses were recorded on a 5-point scale. Higher scores indicated a more positive attitude toward CVD prevention, characterized by a stronger belief in the efficacy of lifestyle modifications for reducing CVD risk. Cronbach’s α of the scale at the time of development^27^ and in this study were 0.81, and 0.92, respectively.

#### Self-efficacy

Participants’ self-efficacy was measured using the Korean version of the Self-Care Self-Efficacy Scale, translated and validated by Lee et al*.*^28^ from the original instrument developed by Becker et al*.*^29^. Self-efficacy refers to an individual’s belief in their ability to efficiently manage health-related practices. It consists of 24 items, rated on a 5-point scale, and representative self-efficacy items include “I can engage in regular exercise that is beneficial to my health” and “I can maintain a balanced diet to support my health.” The self-efficacy score was averaged by summing the scores of the items and dividing by the number of items. Higher scores indicate higher self-efficacy. Cronbach’s α values at the time of instrument development, for the Korean version, and in this study were 0.94, 0.91, and 0.95, respectively.

#### Social support

Social support was measured using the Multidimensional Scale of Perceived Social Support (MSPSS), developed by Zimet et al.^30^ and translated by Park et al.^31^. Social support refers to an individual’s perceived availability and adequacy of emotional and instrumental support from others, including family, friends, and significant others (e.g., partners). The instrument consists of three subscales—support from family, friends, and significant others—with a total of 12 questions, each scored on a 5-point Likert scale. Social support items include: “I can count on my family when I need help” and “I have friends with whom I can share my concerns.” In this study, social support scores were averaged by summing the scores of the items and dividing them by the number of items. Higher scores indicated higher levels of social support. Cronbach’s α values at the time of development^30^, for the version translated by Park et al.^31^, and in this study were 0.92, 0.90, and 0.95, respectively.

#### Intention

Intention refers to an individual’s motivational readiness and conscious plan to perform health- promoting behavior, reflecting the extent to which they are willing to engage in such behavior. In this study, intention was measured using a tool adapted from Jang and Song^32^, who modified the 3-item physical activity intention tool developed by Boudreau and Godin^33^ for individuals with type 2 diabetes. The scale consists of three items rated on a 7-point Likert scale ranging from negative to positive. Sample items include: “I have a plan to engage in health-promoting behavior within the next month” and “I intend to regularly follow my health behavior plans.” The overall score was calculated as the mean of the item scores, with higher scores indicating stronger behavioral intention. Cronbach’s α values at the time of development^33^, in the study by Jang and Song^31^, and in this study were 0.87, 0.91, and 0.95, respectively.

### CVD-preventive health behavior

CVD-preventive health behavior refer to a set of lifestyle practices aimed at reducing CVD risk. This behavior were measured using Kang’s^34^ lifestyle assessment tool. The tool consists of 36 items across six factors: physical activity and weight control; eating habits; alcohol and smoking status; stress; sleep and rest; and medication and medical checkups, each rated on a 4-point scale. Representative items include: “I participate in regular physical activity for a minimum of 30 min per day, at least five days per week” and “I avoid overeating or binge-eating behavior during meals.” The overall score was calculated as the mean of the item scores, with higher scores indicating more favorable CVD-preventive health behavior. Cronbach’s α values at the time of tool development and in this study were 0.92 and 0.93, respectively.

### General characteristics

In this study, the participants’ general characteristics included 12 sociodemographic and health-related variables, based on two previous studies^24,35^. Information on the sociodemographic characteristics of participants was obtained by asking five questions on their age, education level, marital status, occupation, and socioeconomic status. Information on health-related characteristics was obtained by asking six questions on health insurance, subjectively rated health, current chronic disease, time since menopause, alcohol consumption, and smoking status.

### Data collection

Data were collected between June 13 and 18, 2024, using a structured online self-reported questionnaire. Prior to data collection, the questionnaire was pilot tested on five postmenopausal women who met the inclusion criteria to confirm their understanding of the questionnaire content and the time required to complete the questionnaire. The study was conducted online by a panel of professional surveyors who were instructed to check the recruitment advertisement on the homepage and follow the link to the website for further details, including specific study objectives, procedures, and ethical considerations. Women who were willing to participate accessed the online questionnaire through a link. The questionnaire required approximately 15–20 min to complete, and completion of the questionnaire was considered indicative of consent.

### Statistical analysis

The data were analyzed using IBM SPSS/WIN 28.0 and IBM AMOS 28.0 (both IBM Corp., Armonk, NY, USA). The general characteristics of the study participants were analyzed using descriptive statistics and reported using frequencies and percentages for categorical variables, and the relationships between ordinal variables were analyzed using Pearson’s correlation coefficients. Path analysis was performed to test the relationships between CVD knowledge, risk perception, attitudes, self-efficacy, social support, intention, and CVD-preventive health behavior. For the path analysis, a hypothetical model of CVD-preventive health behavior and related variables in postmenopausal women was constructed, and the direct and indirect relationships between variables were examined to assess the goodness of fit. In the presence of multiple mediators, phantom variables were utilized to estimate the specific indirect effects of each mediator, as reported by Macho and Ledermann^36^. These were specified as latent variables without observed indicators, with path coefficients for each targeted mediation pathway fixed at 1.0 and disturbance variables constrained to zero to preserve the original model fit. This method enabled the decomposition and individual estimation of eight unique indirect effects, with statistical significance assessed using bootstrapping with 2000 resamples.

### Ethical approval and informed consent

This study was approved by the Institutional Review Board of Konyang University (Approval No. [2024–05-012]). The survey data were collected using Embrain panel services, a professional online survey platform. All participants had previously provided informed consent to Embrain for the use of their data in academic research. The researchers received fully anonymized data and did not have access to any personal identifying information. All procedures were conducted in accordance with the 1964 Helsinki Declaration and its later amendments or comparable ethical standards.

## Results

### Sociodemographic and health-related characteristics of the participants

A total of 227 women with a mean age of 56.5 years (range 50.0–64.0 years) participated in the study. Of the participants, 164 (72.2%) had college degrees or higher, 176 (77.5%) were married, and 152 (67%) were employed. Among the participants, 137 (60.4%) had a monthly household income of less than 5 million won, 225 (99.1%) had health insurance, 104 (45.8%) reported that their subjective health was moderate, 152 (67.0%) had no chronic diseases, 150 (66.1%) drank alcohol at least once a month, and 219 (96.5%) were nonsmokers. The mean time since menopause was 5.81 years. A statistically significant difference in mean health behavior scores was observed only in relation to subjective health (F = 11.29, *p* < 0.001). Specifically, participants who reported ‘good’ subjective health exhibited significant higher mean health behavior scores than those who reported ‘moderate’ or ‘poor’ health. No other statistically significant differences in mean health behavior scores were observed across any of the sociodemographic or health-related variables (Table 1).

**Table 1:** Participants’ general characteristics (n = 227).

		CVD-preventive health behavior	
Variables	n (%)	Mean ± SD	t or F (*p*)
Age (yr)		56.64 ± 3.34	1.78 (.076)
50–59	180 (79.3)	2.57 ± 0.46	1.51 (.066)
60–64	47 (20.7)	2.69 ± 0.41	
Education			− 1.01 (.318)
≤ High school	63 (27.8)	2.54 ± 0.50	
≥ College	164 (72.2)	2.62 ± 0.44	
Marital status			2.52 (.058)
Unmarried	22 (9.7)		
Married	176 (77.5)		
Divorced	25 (11.0)		
Bereaved	4 (1.8)		
Occupation			1.84 (.067)
Yes	152 (67.0)	2.56 ± 0.44	
No	75 (33.0)	2.68 ± 0.48	
Monthly income (/won)			1.55 (.122)
≤ 5,000,000	137 (60.4)	2.56 ± 0.46	
> 5,000,000	90 (39.6)	2.66 ± 0.44	
Health insurance			− 1.09 (.278)
Yes	225 (99.1)	2.60 ± 0.46	
No	2 (0.9)	2.25 ± 0.12	
Subjective health			11.29 (< .001)
Good^c^	92 (40.5)	2.76 ± 0.41	
Moderate^b^	104 (45.8)	2.49 ± 0.42	
Poor^a^	31 (13.7)	2.46 ± 0.53	
Chronic disease			0.90 (.369)
Yes	75 (33.0)	2.64 ± 0.46	
No	152 (67.0)	2.58 ± 0.45	
Duration of menopause (yr)	5.81 ± 3.86		− 0.52 (.601)
Alcohol drinking			1.04 (.299)
≤ Once/month	150 (66.1)	2.58 ± 0.44	
No	77 (33.9)	2.64 ± 0.48	
Smoking			0.77 (.441)
Yes	8 (3.5)	2.48 ± 0.48	
No	219 (96.5)	2.60 ± 0.45	

SD = standard deviation.

### Descriptive statistics and correlations among the study variables

The coefficients for correlations between variables are shown in Table 2. CVD knowledge showed significant but weak positive correlations with attitudes (*r* = 0.21, *p* = 0.001), self-efficacy (*r* = 0.15, *p* = 0.025), social support (*r* = 0.15, *p* = 0.024), intention (*r* = 0.16, *p* = 0.013), and CVD-preventive health behavior (*r* = 0.14, *p* = 0.034). In contrast, risk perception was not significantly correlated with any of the hypothesized mediators or dependent variables. Regarding the motivational factors, self-efficacy demonstrated moderate positive correlations with social support (*r* = 0.51, *p* < 0.001) and intention (*r* = 0.57, *p* < 0.001). Social support also showed a moderate positive correlation with intention (*r* = 0.45, *p* < 0.001). Finally, CVD-preventive health behavior demonstrated a moderate positive correlation with social support (*r* = 0.42, *p* < 0.001) and strong positive correlation with both self-efficacy (*r* = 0.62, *p* < 0.001) and intention (*r* = 0.62, *p* < 0.001).

**Table 2:** Descriptive statistics and correlation results between variables (n = 227).

Variables	1	2	3	4	5	6	7
	r(*p*)	r(*p*)	r(*p*)	r(*p*)	r(*p*)	r(*p*)	r(*p*)
1. CVD knowledge	1						
2. Risk perception	.01 (.838)	1					
3. Attitude	.21 (.001)	.07 (.279)	1				
4. Self-efficacy	.15(.025)	− .10 (.116)	.10(.130)	1			
5. Social support	.15 (.024)	.00 (.956)	.08 (.253)	.51(< .001)	1		
6. Intention	.16 (.013)	− .02 (.801)	.06 (.340)	.57(< .001)	.45 (< .001)	1	
7. CVD-preventive health behavior	.14 (.034)	− .09 (.158)	.05 (.493)	.62(< .001)	.42 (< .001)	.62 (< .001)	1
Mean ± SD	13.03 ± 2.89	3.34 ± 0.95	3.81 ± 0.46	3.70 ± 0.51	3.50 ± 0.85	4.35 ± 1.44	2.60 ± 0.45
Range (Min–max)		4.75 (1.00–5.75)	3.00 (1.00–4.00)	2.96 (2.04–5.00)	3.92 (1.08–5.00)	6.00 (1.00–7.00)	2.58 (1.36–3.94)

### Model fit indices

To test the relationships between the variables included in the study model, path analysis was conducted using AMOS 28.0. Initially, a theoretical model was constructed as a saturated partial mediation model (df = 0) by connecting all possible paths, including the direct paths, between the independent and dependent variables (Fig. 1). As this model became saturated, global fit indices were no longer interpretable in this context. The model was then modified into a fully mediated model by excluding the non-significant direct paths between the independent and dependent variables to achieve a more parsimonious structure. The final modified model demonstrated an excellent fit to the data (χ^*2*^ = 1.17, df = 1, *p* = 0.279; CMIN/df = 1.17; RMSEA = 0.00; GFI = 1.00; CFI = 1.00; and TLI = 1.04).

### Direct effects on CVD-preventive health behavior

To test the influence of the relationship between the study variables, the path coefficients and statistical significance of each path were examined. In the final model, six of the 12 paths between variables were statistically significant (Table 3).

**Table 3:** Results of the path analysis showing path estimates of the modified model (n = 227).

Path	*β*	B	SE	C.R	*p*
CVD Knowledge → Attitude	.21	.03	.01	3.30	< .001
CVD Knowledge → Self-efficacy	.15	.03	.01	2.30	.021
CVD Knowledge → Social support	.15	.04	.02	2.27	.023
CVD Knowledge → Intention	.17	.08	.03	2.51	.012
Risk perception → Attitude	.07	.03	.03	1.07	.286
Risk perception → Self-efficacy	− .11	− .06	.04	− 1.63	.103
Risk perception → Social support	− .01	− .01	.06	− 0.09	.930
Risk perception → Intention	− .02	− .03	.10	− 0.29	.771
Attitude → CVD-preventive health behavior	− .02	− .02	.05	− 0.42	.673
Self-efficacy → CVD-preventive health behavior	.37	.33	.06	5.95	< .001
Social support → CVD-preventive health behavior	.06	.03	.03	1.02	.309
Intention → CVD-preventive health behavior	.38	.12	.02	6.41	< .001

The paths from the first independent variable, CVD knowledge, to attitudes (β = 0.21, *p* < 0.001), self-efficacy (β = 0.15, *p* = 0.021), social support (β = 0.15, *p* = 0.023), and intention (β = 0.17, *p* = 0.012) were significantly positive. Higher levels of CVD knowledge were associated with higher levels of attitudes, self-efficacy, social support, and intention. However, the paths from risk perception to attitudes, self-efficacy, social support, and CVD-preventive health behavior were not significant, suggesting that risk perception did not significantly affect attitudes, self-efficacy, social support, or health behavior. When the effects of the mediator variables, attitudes, self-efficacy, social support, and intention, on the dependent variable (CVD-preventive health behavior) were examined, the paths from self-efficacy (β = 0.37, *p* < 0.001) and intention (β = 0.38, *p* < 0.001) to CVD-preventive health behavior were significantly positive (Table 3). The final model explained 48.7% of the variance in CVD-preventive health behavior (R^2^ = 0.487). Self-efficacy and health behavior intention were significant direct predictors of CVD-preventive health behavior.

### Indirect effects on CVD-preventive health behavior

We used bootstrap testing to test the indirect effects of CVD knowledge and risk perception on health behavior through the four parameters. Owing to multiple mediators, phantom variables were created to test the exact indirect effect of each mediator.

In this study’s path model, the relationship between CVD knowledge, risk perception, and CVD-preventive health behavior could be explained by several indirect paths. The indirect effect of CVD knowledge on CVD-preventive health behavior could be explained through self-efficacy (β = 0.055, *p* = 0.040) and the intention to engage in health behavior (β = 0.063, *p* = 0.018) (Table 4).

**Table 4:** Indirect effects on CVD-preventive health behavior (n = 227).

Path	*β*	B	95% CI	*p*
CVD knowledge → Attitude → CVD-preventive health behavior	− .004	− .001	− .006 ~ .002	.448
CVD knowledge → Self-efficacy → CVD-preventive health behavior	.055	.009	.001 ~ .020	.040
CVD knowledge → Social support → CVD-preventive health behavior	.009	.001	− .001 ~ .006	.169
CVD knowledge → Intention → CVD-preventive health behavior	.063	.010	.002 ~ .022	.018
Risk perception → Attitude → CVD-preventive health behavior	− .001	− .001	− .009 ~ .002	.389
Risk perception → Self-efficacy → CVD-preventive health behavior	− .039	− .019	− .045 ~ .001	.063
Risk perception → Social support → CVD-preventive health behavior	.000	− .001	− .016 ~ .016	.953
Risk perception → Intention → CVD-preventive health behavior	− .007	− .003	− .030 ~ .020	.770

B = unstandardized path coefficient; CI = Confidence interval; *β* = standardized path coefficient.

## Discussion

This study presents a novel exploration of the factors influencing CVD-preventive health behavior in postmenopausal women using the I-Change theoretical framework. The final model indicated that CVD-preventive health behavior were significantly associated with CVD knowledge, self-efficacy, and intention. Notably, CVD knowledge was not directly associated with CVD-preventive health behavior but was indirectly related through self-efficacy and intention, suggesting their crucial mediating roles within the proposed model. These findings highlight the importance of considering both self-efficacy and intention when designing interventions to promote CVD-preventive health behavior change in this population.

### Self-efficacy

Self-efficacy was found to be significantly associated with CVD-preventive health behavior and acted as a mediator in the relationship between CVD knowledge and CVD-preventive health behavior. This finding is consistent with Bandura’s Social Cognitive Theory^37^, which identifies self-efficacy as a key determinant of CVD-preventive health behavior. Previous studies have also demonstrated the importance of self-efficacy in maintaining healthy lifestyle habits^38^ and its mediating role in the relationship between knowledge and self-management behavior^39^.

In the present study, self-efficacy showed a significant indirect pathway between CVD knowledge and CVD-preventive health behavior. However, given the cross-sectional design, this finding should be interpreted as a statistical association within the proposed model rather than as evidence of a causal mechanism.

One possible explanation for this pattern may be related to contextual factors specific to postmenopausal Korean women. For example, previous literature suggests that women in this age group may prioritize family responsibilities over their own health, which could influence the extent to which knowledge alone leads to behavior change^40^. In this context, self-efficacy may play an important role in enabling individuals to engage in CVD-preventive health behavior. However, this interpretation should be considered as a plausible contextual explanation rather than a direct finding of the present study. These findings suggest that interventions aimed at improving cardiovascular health should not only focus on increasing knowledge but also on enhancing self-efficacy through strategies such as goal setting, positive reinforcement, and motivational support.

### Intention

Intention was also significantly associated with CVD-preventive health behavior and demonstrated a mediating role in the relationship between CVD knowledge and CVD-preventive health behavior. This finding aligns with the Theory of Planned Behavior, which identifies intention as a key proximal determinant of behavior^41^.

The results suggest that knowledge alone may not be sufficient to influence health behavior without a corresponding intention to act. This may be related to the comprehensive nature of CVD-preventive health behavior assessed in this study, which included multiple domains such as diet, exercise, and lifestyle management. Compared with studies focusing on a single health behavior^42^, broader behavioral outcomes may require stronger motivational components to translate knowledge into action. Similar patterns have been reported in patients with heart failure, where knowledge alone did not directly influence self-management behavior^43^.

In the context of postmenopausal Korean women, previous studies have suggested that intention toward CVD-preventive health behavior may be relatively low, particularly for physical activity^44^. This may contribute to a gap between awareness and actual behavior. To address these issues, tailored interventions should focus not only on enhancing CVD knowledge but also on strengthening intention by emphasizing the long-term benefits of CVD-preventive health behavior, both for personal well-being and the capacity to care for their families. In addition, qualitative studies exploring the factors influencing intention in this group can provide valuable insights. By identifying specific barriers and facilitators, this study can guide the design of culturally sensitive interventions to strengthen intention and overcome unique challenges.

These results highlight the importance of strengthening behavioral intention in intervention strategies, for example by emphasizing the benefits of health behavior and supporting individuals in setting clear and achievable goals.

### Risk perception

Risk perception was not significantly associated with any of the variables in the model, including CVD-preventive health behavior. This finding contrasts with previous studies that have identified risk perception as an important factor in promoting health behavior^12,43^.

Several factors may explain this discrepancy. First, the characteristics of the study sample may have influenced the results. Most participants were relatively young postmenopausal women with few underlying health conditions and relatively good perceived health status. As risk perception is often associated with perceived vulnerability, lower perceived risk in this group may have reduced its influence on CVD-preventive health behavior.

Second, the measurement tool used to assess risk perception may have limited its sensitivity in this population. Items related to the likelihood of experiencing a heart attack or related worry may have been less applicable to individuals who perceive themselves as generally healthy.

Given these considerations, the lack of association observed in this study should be interpreted with caution. Further research is needed to examine how risk perception operates across different populations and measurement approaches.

### Strength and limitations

The strength of this study is that it examined the associations among CVD knowledge, risk perception, motivation factors, and CVD-preventive health behavior using the I-Change model to provide both theoretical insights and practical implications. Additionally, to examine the indirect effects of multiple mediators, phantom variables were created, allowing for a more precise estimation of specific indirect pathways. However, several limitations of this study should be considered. First, because data were collected through an online survey, the sample tended to include participants with relatively higher educational attainment, while individuals with limited Internet access may have been underrepresented. This recruitment approach may have introduced selection bias and limited the representativeness of the sample. Although participants were recruited from various regions in South Korea, the degree to which the sample reflects the broader population remains uncertain. Second, the cross-sectional nature of the study restricts the ability to draw causal conclusions among the study variables. Accordingly, the identified associations should be interpreted as correlational rather than causal, and caution is warranted when interpreting the findings. Future research employing longitudinal or experimental designs is recommended to further examine the proposed relationships and underlying mechanisms. Third, the relatively modest sample size may limit the generalizability of the findings. Studies involving larger and more heterogeneous populations are needed to strengthen the external validity of the results. Finally, this study relied on self-reported survey data and lacked objective cardiovascular health indicators, which may limit the model’s comprehensiveness and introduce recall bias. Although additional analyses controlling for age, income, and education level yielded consistent mediation pathways, model fit declined with the inclusion of these covariates. Therefore, the final model was specified without them to maintain parsimony. Future research should incorporate objective cardiovascular health indicators to further validate these findings.

## Conclusion

The findings of this study suggest that self-efficacy and intention play important mediating roles in the relationship between CVD knowledge and CVD-preventive health behavior among postmenopausal Korean women. CVD knowledge was not directly associated with CVD-preventive health behavior but was indirectly related through motivational factors. These findings suggest that effective CVD prevention interventions should extend beyond the provision of information and incorporate strategies to enhance self-efficacy and strengthen behavioral intention. Such approaches may support the promotion of CVD-preventive health behavior in this population.

## Data Availability

The datasets generated and/or analyzed during the current study are not publicly available due to contractual restrictions with the business panel provider but are available from the corresponding author on reasonable request.
